# Factors influencing gastroenterology specialist trainees’ satisfaction with the regional speciality educational programme

**DOI:** 10.15694/mep.2017.000017

**Published:** 2017-01-30

**Authors:** Ian Beales

**Affiliations:** 1 University of East Anglia

**Keywords:** gastroenterology, curriculum design, teaching organisation, postgraduate

## Abstract

This article was migrated. The article was marked as recommended.

All post-graduate training programmes in the United Kingdom follow relevant competency-based curricula. Whilst much of trainees learning occurs during the day-day activities of their training posts, all training programmes support their trainees with a formal taught educational programme, usually based on full or half-days of education for all trainees on that training scheme. The factors which influence trainees learning and satisfaction with these educational programmes have not been examined. The factors associated with increased trainee- perception of high teaching quality and the effect on practice from all regional teaching sessions over a 6 year period were examined in the East of England Gastroenterology training scheme. There was a very strong correlation between perceived teaching skills of the educator and the scores for effect on practice. Teacher and curriculum factors associated with higher scores for effect on practice were examined by unconditional logistic regression. Overall two teacher-related factors were independently associated with highest scores for effective teaching: having a formal post-graduate educational qualification and having published a paper on the relevant topic in the last 12 months. No curriculum-related factors were related to the perceived qualify of the teaching session. These data provide some insight into factors that should be considered when designing regional speciality teaching programmes and emphasize that educational qualifications appear to be a very important marker of high-quality teaching.

## Introduction and Objectives

In the United Kingdom, post-graduate Gastroenterology specialist training, like all specialities is currently directed by a competency-based curriculum. Whilst most education and training to support the trainees is work-based, there are also regular formal educational sessions for all trainees, which complement and inform the more practical medical and technical skills usually developed as part of a training post (JRCPT 2010). This sessions are formal educational events involving all trainees in any one regional training programme. At present, this reginal teaching programme is a rolling programme covering the whole gastroenterology curriculum in 30 days over 2.5 years. Although there is a considerable literature on both methods of delivering undergraduate medical education and teaching in general, there are no data available to inform the construction of such specific postgraduate teaching programmes. In contrast, there is significant literature on clinical and opportunistic teaching (Harden & Crosby, 2000, Spencer 2003, Gordon 2003) and well-established training pathways in gastrointestinal endoscopy (JAG 2016) . This study aimed to explore the factors associated with increased satisfaction amongst specialist trainees with the educational sessions delivered within the regional educational sessions, to inform design of future educational programmes.

## Design

The East of England Regional Gastroenterology Teaching programme over 60 educational sessions 2010-2104 was assessed. After each session trainees rated teaching sessions using a 10-part Likert scale on both 'how much the session influenced my practice,' and 'teaching skills employed'. Mean scores for each teaching session were correlated with factors related to topic or teacher.

## Setting

East of England Gastroenterology Training Scheme 2010-2014.

## Participants

Gastroenterology specialist trainees, 47 in total over the 5-year cycle. Teaching was provided by Gastroenterology specialists and trainees as well as those from medical and non-medical backgrounds as related to the curriculum, including gastrointestinal surgeons, radiologists, pathologists and microbiologists, specialist nurses, dieticians and other professions with a stake in the training of gastroenterologists.

## Main outcome measures

Factors associated with increased trainee satisfaction with teaching received. Adjusted odds ratio of factors associated with sessions in the top tertile compared to the bottom tertile were calculated using unconditional logistic regression.

## Results

Overall 246 teaching sessions were included. There was a strong correlation between scores for 'teaching skills' and 'influence on practice,' (Pearson's r = 0.917)(p<0.001), although only 'influence on practice' was used for further analysis of satisfaction. Table 1 shows the adjusted odds ratios of teacher and topic factors associated with scoring in the top tertile for satisfaction compare to the bottom tertile.

Overall the strongest association with satisfaction was teaching provided by a teacher with a formal post-graduate teaching qualification (MAcadMedEd, FHEA, MSc or PGCert), independent of any other factor related to the teacher or topic. In contrast, the standard clinical TTT course (teaching teachers to teach course, which is undertaken by many senior trainees and consultants) had no relationship to satisfaction. There was a strong association between increased satisfaction and the teacher having published a paper on the relevant topic within 12 months, the strength of this association decreased with increasing time since last relevant publication such that 36-60 months had no association and having published a relevant paper > 60 months previously was associated with low satisfaction scores. Overall consultants scored higher than trainees, and gastroenterology specialists higher than other specialities (e.g. microbiology or epidemiology) or those from a non-medical background. There was no difference in satisfaction scores related to area of curriculum.

**Table 1:** ***Teacher and topic related factors in postgraduate gastroenterology teaching associated with highest trainee satisfaction*** *.* Adjusted odds ratios and 95% confidence intervals for high scoring (top tertile) compared to low scoring (bottom tertile).

**Table:** 

	**Adjusted odds ratio**	**95% confidence intervals**
**Male**	1.05	0.55 - 1.99
**Female**	0.95	0.50 - 1.79
**Consultant**	3.11	1.60 - 6.39
**Trainee**	0.70	0.36 - 1.31
**Other grade/background (nurse etc)**	0.39	0.12 - 0.97
**Higher degree**	5.55	2.61 - 12.02
**No higher degree**	0.18	0.08 - 0.38
**Formal teaching qualification**	89.3	12.2 - 672.0
**Regional TTT course**	1.34	0.85 - 3.37
**University Hospital (undergraduate teaching/research/referral centre) appointment**	1.69	0.85 - 2.44
**District general hospital appointment **	0.67	0.59 - 2.44
**Publication on subject < 12 months previously**	21.1	3.63 - 452.2
** Publication on subject 12-36 months previoulsy**	3.83	1.46 - 10.94
**Publication on subject 36 - 60 months previously**	0.90	0.47 - 1.73
**Publication on subject > 60 months previously**	0.10	0.05 - 0.55
**Gastroenterologist**	3.80	1.84 - 7.91
**Surgeon**	1.56	0.67 - 3.71
**Other speciality**	0.48	0.24 - 1.08
**Topic: upper GI**	0.86	0.40 - 1.85
**Topic: liver & biliary tree**	0.87	0.45 - 1.80
**Topic: small bowel and nutrition**	1.10	0.45 - 2.72
**Topic: lower GI**	1.56	0.75 - 3.42

## Discussion

The two clearest independent predictors of increased trainee satisfaction were teacher-related: having a formal teaching qualification and having published a paper recently on the topic. It would be wise to consider these when designing future teaching programmes. It is not advisable to exclude factors related to lower scores from future programmes, indeed it is probably desirable to encourage trainees to teach each other, as part of their personal and professional development but effective formative feedback should be provided.

Despite the existence of a clearly defined competency-based curriculum and a regular series of appraisals via the annual record of competency progression (ARCP) progress, there is minimal evidence and guidance on how post-graduate programme directors should develop and construct the regional-teaching programme to support the knowledge of the trainees. It is suspected that choices are made based on past-practice and tradition, teacher interest and availability as well as reputation both for research and (hopefully) teaching ability.

The evidence from this study does suggest that gastroenterology trainees appear to gain most from sessions run by either teachers with a teaching qualification or those that have published a paper on the relevant topic in the previous 12 months. These factors can be used to optimise the design of a teaching programme. Although having a session led by a teacher with a teaching qualification, was strongly related to learner satisfaction, it is not clear at present whether the teaching qualification is merely a marker of interest and commitment to teaching or whether the process of obtaining the qualification, clearly positively influences teaching practices and learning in this particular context.

These data are directly related to gastroenterology trainees and their learning. There are no comparative studies in other specialities and it will be very interesting to evaluate similar factors in both other physician-specialities (such as cardiology or respiratory medicine) and other specialities such as surgery or histopathology.

## Take Home Messages

Although regional training programme teaching sessions form a core part of postgraduate training in the United Kingom, there is a paucity of data concering the construction and evaluation of such educational sessions. In this study, no curriculum-related factors where associated with increased or decreased learner satisfactioon with the sessions. However several teacher-related factors were associated with Recent (within 12 months) publication of a paper on the relevent topic was also independanrly associated with trainees' pereived effectiveness of the teaching session, interesting this effect declined rapidly with time as the interval to last relevent publication increased. Overall tutor-related factors seem dominant affecting percieved qulaity and usefulness of the formal teaching sessions. These factors can be used in designing future teaching programmes.

## Notes On Contributors

Dr Ian Beales is Clinical Reader in Gastroenterological Pharmacology at University East Anglia Medical School and Honorary Consultant Gastroeterology at Nofolk and Norwich University Hospital. He is currently Director of the East of England Endoscopy Trainng Centre and Chair and previously was Training Porgramme Director for Gastroenterology in East of England. He has broad interest across clincial gastroenterology & edication including skills training and assessment methods.

## Bibliography/References

Gordon J. One to one teaching and feedback. BMJ 2003 326:543. https://doi.org/10.1136/bmj.326.7388.543

Harden R M, Crosby J R. AMEE Education Guide No 20: The good teacher is more than a lecturer-the twelve roles of the teacher. Medical Teacher 2000;22:334-47. https://doi.org/10.1080/014215900409429

http://www.thejag.org.uk/downloads/JAG%20Certification%20for%20trainees/OGD%20application%20criteria%20and%20process.pdf (accessed 24/1/2017) 

https://www.jrcptb.org.uk/sites/default/files/2010%20Gastroenterology%20%28amendment%202013%29_0.pdf (accessed 24/01/2017) 

Spencer J. Learning and teaching in the clinical environment. BMJ 2003 326:591. https://doi.org/10.1136/bmj.326.7389.591

## Appendices

## Declarations

The author has declared the conflicts of interest below.

The author is a member of the Academy of Medical Educators and Fellow of the Higher Education Academy.

