# Supplementary material for: Factors influencing gastroenterology specialist trainees’ satisfaction with the regional speciality educational programme
Source: MedEdPublish (2016). 2017 Jan 30;6:17. [Version 1] doi: 10.15694/mep.2017.000017 (PMC10885219; doi:10.15694/mep.2017.000017)
Supplement: Peer review history

# Reviewer report: Reviewer response for version 1

**DOI:** 10.21956/mep.19433.r28583

**Published:** 2017-02-01

**Authors:** Gert Van Zyl

**Affiliations:** 1 University of the Free State

This review has been migrated. The reviewer awarded 3 stars out of 5 A very interesting short article. The ago old battle between being and educationalist and a clinician; teaching skills and "influence on practice" and teaching versus learning is again demonstrated. The outcomes from the research is interesting. It is not mentioned in the piece if the trainees knew the formal qualification of the trainers when they completed the research and if this might have influenced the outcome? Nothing more to add to the previous reviews but to say that the work has some typing errors - example - Under Take home message- satisfaction - that can be corrected. Support the view that the work can grow as a research initiative.

Reviewer Expertise:

NA

No decision status is available

# Reviewer report: Reviewer response for version 1

**DOI:** 10.21956/mep.19433.r28585

**Published:** 2017-01-31

**Authors:** Mohammed Elhassan

**Affiliations:** 1 SAMP

This review has been migrated. The reviewer awarded 4 stars out of 5 As a faculty in internal medicine residency program in US, I think I learned something from this paper. Although, more data are still needed to go into the depth of the reasons behind the interesting results of the survey and to examine the generalizability of its results to other specialties, it does make sense that teachers with teaching qualifications and those with recent publication in the topic they teach receive the highest learners satisfactions. Teachers should keep that in mind if they want to improve their teaching experience. It is quite interesting to notice that the time of the publications made such difference in the results.

Reviewer Expertise:

NA

No decision status is available

# Reviewer report: Reviewer response for version 1

**DOI:** 10.21956/mep.19433.r28586

**Published:** 2017-01-31

**Authors:** Richard Hays

**Affiliations:** 1 James Cook University

This review has been migrated. The reviewer awarded 3 stars out of 5 The only comment I would add to those of the other reviewers is a mild concern that learner satisfaction seems higher for teachers with a recent publication on the content of their teaching. This is a good thing, from an academic perspective, and should be pursued by clinicians outside of major centres, but there are disadvantages faced by researchers outside of major centres in participating in research and publishing. Such a correlation might also further encourage regional hospitals to emulate larger academic centres rather than find their own place in clinical and learning services. Could the recent publication be a proxy for something else, such as passion for a topic that was recently studied/reviewed? Fostering scholarship in regional centres may be the key to success.

Reviewer Expertise:

NA

No decision status is available

# Reviewer report: Reviewer response for version 1

**DOI:** 10.21956/mep.19433.r28584

**Published:** 2017-01-30

**Authors:** John Cookson

**Affiliations:** 1 University of Worcester

This review has been migrated. The reviewer awarded 4 stars out of 5 An interesting paper but as you say it is likely that those with a teaching qualification are more interested in teaching. Those with a recent publication are probably seen as more authoritative but are also those most likely to be seen independently as leaders in the field and as postgraduate examiners. You may be seeing a 'halo effect'.

Reviewer Expertise:

NA

No decision status is available

# Reviewer report: Reviewer response for version 1

**DOI:** 10.21956/mep.19433.r28587

**Published:** 2017-01-30

**Authors:** Trevor Gibbs

**Affiliations:** 1 AMEE

This review has been migrated. The reviewer awarded 4 stars out of 5 An interesting, although relatively short, piece of research that I believe conveys an important message.I believe it describes the difference between teaching and learning- teaching being a one-way process of imparting information, learning being a two-way process that envelops teaching into an environment of collaboration between student(s) and tutor, in a motivational and positive / passionate environment. Although many of the peripheral and central clinical teachers have attended some form of teaching the teachers course, they appear not to have extended this into practice, reflected upon their approaches and methodologies and are really teaching with that true passion of wanting the students to learn. I would like to see this piece of research grow into a wider piece that explores in greater depth what are the qualities required of a "good teacher" and then expand this into their effects - short and long time- upon future trainees.

Reviewer Expertise:

NA

No decision status is available
